# HIV-1-Based Virus-like Particles that Morphologically Resemble Mature, Infectious HIV-1 Virions

**DOI:** 10.3390/v11060507

**Published:** 2019-06-02

**Authors:** Christopher A. Gonelli, Georges Khoury, Rob J. Center, Damian F.J. Purcell

**Affiliations:** 1 Department of Microbiology and Immunology, Peter Doherty Institute for Infection and Immunity, The University of Melbourne, Melbourne, Victoria 3000, Australia; c.gonelli@student.unimelb.edu.au (C.A.G.); georges.khoury@unimelb.edu.au (G.K.); rob.center@burnet.edu.au (R.J.C.); 2 Viral Entry and Vaccines Laboratory, Disease Elimination, Burnet Institute, Melbourne, Victoria 3004, Australia

**Keywords:** HIV-1, virus-like particle, VLP, mature-form, particle assembly

## Abstract

A prophylactic vaccine eliciting both broad neutralizing antibodies (bNAbs) to the HIV-1 envelope glycoprotein (Env) and strong T cell responses would be optimal for preventing HIV-1 transmissions. Replication incompetent HIV-1 virus-like particles (VLPs) offer the opportunity to present authentic-structured, virion-associated Env to elicit bNAbs, and also stimulate T cell responses. Here, we optimize our DNA vaccine plasmids as VLP expression vectors for efficient Env incorporation and budding. The original vector that was used in human trials inefficiently produced VLPs, but maximized safety by inactivating RNA genome packaging, enzyme functions that are required for integration into the host genome, and deleting accessory proteins Vif, Vpr, and Nef. These original DNA vaccine vectors generated VLPs with incomplete protease-mediated cleavage of Gag and were irregularly sized. Mutations to restore function within the defective genes revealed that several of the reverse transcriptase (RT) deletions mediated this immature phenotype. Here, we made efficient budding, protease-processed, and mature-form VLPs that resembled infectious virions by introducing alternative mutations that completely removed the RT domain, but preserved most other safety mutations. These VLPs, either expressed from DNA vectors in vivo or purified after expression in vitro, are potentially useful immunogens that can be used to elicit antibody responses that target Env on fully infectious HIV-1 virions.

## 1. Introduction

Following the discovery of human immunodeficiency virus type 1 (HIV-1) almost thirty-five years ago and its role as the etiological agent of acquired immunodeficiency syndrome (AIDS), the resulting pandemic has become one of the most significant infectious disease outbreaks in recent human history. The introduction of combined anti-retroviral therapy (cART) more than two decades ago has delayed disease progression and reduced mortality in patients from many developed countries [1]. Yet, developing regions continue to be adversely impacted by the enormous social and economic costs of the global epidemic, which warrants research investigating the prevention of HIV as an urgent priority.

An effective prophylactic vaccine for HIV-1 likely requires the elicitation of broadly neutralizing antibodies (bNAbs) against conserved epitopes on the envelope glycoprotein complex (Env), given the high amino acid variability of Env in circulating isolates [2]. Initial efforts at eliciting bNAbs focused on recombinant monomeric gp120, due to the labile, non-covalent interactions between the Env receptor-binding (gp120) and membrane-spanning (gp41) domains [3,4]. Such approaches were met with limited success; while the gp120 was immunogenic, it failed to raise neutralizing antibody responses in animal models or humans [5,6]. However, the only human vaccine trial designed to demonstrate protection from infection, albeit modest and transient, the RV114 phase III vaccine trial, also utilized monomeric gp120 as part of the vaccination regime [7]. In the RV114 trial, the participants were primed with a live recombinant, non-replicating Canarypox vector encoding a membrane-bound gp160 prior to boosting with recombinant gp120 proteins. This suggests the initial immune response against membrane-bound Env primed humoral responses that were capable of binding similarly membrane-bound, trimeric Env on HIV-1 virions and that could block viral infection. Recombinant, trimeric gp120 fused to the gp41 ectodomain has been generated by either abolishing the native cleavage site (UNC gp140) [8,9] or by introducing disulfide linkages and stabilizing modifications (SOSIP gp140) [10,11]. These oligomers have demonstrated enhanced immunogenicity when compared to monomeric gp120 protein and induced antibody responses that are capable of neutralizing various circulating strains of HIV-1 [12,13,14,15].

A strategy that may improve the elicitation of bNAbs involves the vaccination with Env presented in a membrane-bound context, such as on the surface of a virus-like particle (VLP). Vaccines utilising HIV-1 VLPs were found to induce strong antibody responses in small animal models and macaques [16,17]. HIV-1 VLPs may provide several advantages over conventional vaccination with recombinant Env proteins, as the Env displayed on HIV-1 VLPs is likely to be presented in a trimeric conformation [18], which closely mimics that on the native virion targeted by a vaccine. Additionally, the innate and adaptive arms of the immune system seem to respond particularly well to virus-sized antigens due to the efficient drainage of these particles into lymph nodes and subsequent antigen sampling by antigen-presenting cells [19].

Many of the HIV-1 VLP immunogen approaches have utilized Gag-only expression vectors to mediate the expression of VLPs that ultimately mimic immature, non-infectious virions [20,21,22,23]. This is due to the absence of the viral Pol, which encodes the protease (PR) domain that normally facilitates proteolytic cleavage of the Gag polyprotein and the maturation of virions into their infectious form [24,25]. While the absence of Pol allows for high yields of VLPs with Env being incorporated into the particle membrane, the morphology of immature HIV-1 virions does not permit the lateral movement and clustering of Env on the virion membrane, resulting in individual Env spikes that are sparsely distributed across the particle surface [26]. When coupled with the approximately ten copies of Env trimers incorporated into individual particles [27], this would likely limit the ability of B cell antibody receptors to be engaged and activated to initiate an effective humoral response.

The disadvantage of HIV-1 VLPs that resemble mature virions is that they are typically expressed from vectors encoding a HIV-1 Env-deficient, particle-forming vector that retains the ability to reverse transcribe and integrate their genetic material into a host cell genome [16,28,29]. Viral inactivation with aldrithiol-2 (AT-2) can be used to ensure that the particles are non-infectious [30] and the lack of a functional Env sequence in the VLP genome ensures that only a small number of replication-competent particles would be initially present [31]. However, the need to inactivate the VLPs adds an additional processing step during particle production and it limits the VLPs to in vitro expression from cell lines transfected with at least two plasmids and subsequent injection of purified particles in a prophylactic vaccine setting. Although several chromatographic technologies have been developed to more easily separate VLPs from contaminating cellular microvesicles and exosomes in cell culture supernatant [32,33], large-scale in vitro production of VLPs is often difficult [34]. Moreover, many cellular proteins are incorporated into HIV-1 viral particles, which will persist on purified VLPs [35,36]. Therefore, immunogenicity studies may likely be prone to significant cross-species immune responses depending on the cell line species that are used for VLP production and small animals employed for vaccination. These cross-species responses could be circumvented by the expression of VLPs in vivo from nucleic acid vaccine vectors. The efficient expression of VLPs from such a strategy would likely require the use of a single-plasmid vector that does not produce infectious viral particles.

Here, we developed single-plasmid, HIV-1 VLP vectors for the production of non-infectious particles that incorporate Env and resemble fully-infectious, mature HIV-1 virions. These vectors were adapted for the efficient and safe particle expression from a previously developed chimeric VLP vector expressing AE clade Gag/Pol and B clade Env polyproteins [37], which were derived from HIV-1 DNA T cell vaccine vectors that were designed for maximal safety and were used in human phase I/IIa trials [38,39]. We found that the functional site deletions within the reverse transcriptase (RT) domain of the original VLP vector led to the production of immature and irregularly sized VLPs. The restoration of these deletions gave rise to VLPs with similar morphology to fully infectious, mature virions. However, the deletion of the entire RT domain yielded similarly mature but non-infectious VLPs that were capable of Env-mediated fusion with CD4+ T cell lines.

## 2. Materials and Methods

### 2.1. Cells, Enzymes, Oligonucleotides and Chemicals

Adherent HEK 293T cells were maintained in high glucose DMEM that was supplemented with 1× GlutaMAX™-I and 10% (*v/v*) heat-inactivated FBS (Thermo Fisher Scientific, IL, USA) (growth medium). Suspension CEM.NKR CCR5+ cells were maintained in RPMI-1640 medium supplemented with 1× GlutaMAX™-I and 10% (*v/v*) heat-inactivated FBS (Thermo Fisher Scientific). All of the PCRs were performed using Phusion^®^ DNA polymerase (NEB, MA, USA) and enzymes used for plasmid construction were purchased from NEB. All of the oligonucleotides and chemicals were obtained from Sigma, unless otherwise specified.

### 2.2. Monoclonal and Polyclonal Antibodies

The monoclonal antibodies (mAbs) and polyclonal sera that were obtained from the NIH AIDS reagent repository (ARP) included anti-p24 mAb #24-2 [40], anti-Env polyclonal serum DV-012 [41], and anti-Env mAbs 17b [42], 35O22 [43], 447-52D [44], F105 [45], F240 [46], PGT121 [47], VRC01 [48], and Z13e1 [49]. Anti-Env mAbs 2F5, 2G12, 4E10, PG9, and PG16 were purchased from Polymun (Vienna, Austria). Anti-p24 mAb BC1071 and sheep polyclonal antibodies anti-p24 (D7320) and anti-gp120 (D7324) were purchased from Aalto Bio Reagents (Dublin, Ireland).

### 2.3. Recombinant gp120 and p24

The production of recombinant gp120 that was encoded by the Env from NL(AD8) [50] (herein referred to as AD8 strain) utilized a stable cleavable gp140-expressing HeLa cell line. The cell line was generated and the Env-containing supernatant was produced, as described previously [37]. The gp120 was purified from culture supernatant by lentil lectin affinity chromatography, as described previously [51]. The purified gp120 was concentrated and further purified by size exclusion chromatography while using a Superdex™ 200 16/600 pg with an ÄKTAprime plus liquid chromatography system (GE Healthcare Life Sciences, Little Chalfont, UK). The protein was injected into the column that was equilibrated with PBS and the flow rate was 1 mL/min. Fractions containing monomeric gp120 were pooled and concentrated. Recombinant p24 that was encoded by the Gag from isolate BH10 was purchased from Aalto Bio Reagents.

### 2.4. Plasmids

The full-length, molecular clone expression plasmids p93TH253.3 [52], pNL4-3 [53], and pNL (AD8) [50] were obtained from the NIH ARP. The EGFP expression vector pCMV-EGFP was constructed previously [37]. The AD8 Env expression plasmid pCMV-AD8 was described previously [54].

The AEB VLP expression plasmid pCMV-AEB was generated by subcloning the NotI/BclI fragment from the previously described pVLP [37] into pcDNA3.1(-) (Thermo Fisher Scientific) while using the NotI and BamHI sites. For pCMV-AEB.IN, a functional integrase (IN) domain was restored by amplifying the domain from p93TH253.3 and introducing the flanking EcoRI sites and a premature stop codon in the vif open reading frame (ORF) (Odp.2856 & Odp.2857, see Supplementary Table S1 for these and subsequent primer sequences). The fragment was then ligated into pCMV-AEB while using the EcoRI site.

The plasmids pCMV-AEB.NC and pCMV-AEB.NC.IN were generated by splicing a functional nucleocapsid (NC) domain that was amplified from p93TH253.3, with flanking gag and gag-pol fragments from pCMV-AEB and pCMV-AEB.IN by overlap-extension PCR. These spliced fragments were then ligated into pCMV-AEB and pCMV-AEB.IN, respectively, using NotI and EcoRI.

The pol region was removed from pCMV-AEB by amplifying the gag and introducing a 3′ EcoRI site (Odp.0241 & Odp.1067) before subcloning this fragment back into the vector using NotI and EcoRI to create pCMV-AEp55B. A Tat-deficient AEB expression plasmid, pCMV-AEB.Δtat, was generated by linearizing pCMV-AEB at the MfeI site within the tat ORF, end-filling with Klenow, and then blunt-end re-ligation.

A truncated 5′ LTR fragment (5LTRΔU3) was amplified from p93TH253.3, introducing a novel 5′ flanking NotI site and containing an existing 3′ SpeI site (Odp.3032 & Odp.0479). The plasmids pCMV-5LTRΔU3-AEB.NCΔtat and pCMV-5LTRΔU3-AEB.NC.INΔtat were then created by simultaneously ligating the PCR NotI/SpeI fragment with a SpeI/EcoRI fragment from pCMV-AEB.NC or pCMV-AEB.IN, respectively, into pCMV-AEB.Δtat while using the NotI and EcoRI sites.

A functional RT domain was introduced by ligating the SpeI/PvuII fragment from p93TH253.3 into pCMV-5LTRΔU3-AEB.NC.Δtat and pCMV-5LTRΔU3-AEB.NC.INΔtat for pCMV-5LTRΔU3-AEB.NC.RTΔtat and pCMV-5LTRΔU3-AEB.NC.RT.INΔtat, respectively. The plasmid pCMV-5LTRΔU3-AEB.RT.Δtat was generated by replacing the NotI/SpeI and SpeI/BsaWI fragments with those from pCMV-5LTRΔU3-AEB.NC.Δtat and pCMV-AEB, respectively. A similar method was used to create pCMV-AEB.NC.RT.Δtat, except that the respective fragments were from pCMV-AEB and pCMV-AEB.NC. The plasmid pCMV-AEB.RT.Δtat was generated by replacing the NotI/BsaWI fragment with that from pCMV-AEB. The plasmid pCMV-AEB.RT was then created by replacing the EcoRI/BlpI fragment of pCMV-AEB.RT.Δtat with that from pCMV-AEB.

Combinations of one or two of the three RT mutations that were present in pCMV-AEB were introduced into pCMV-AEB.RT.Δtat by splicing three PCR fragments covering the target RT regions that were amplified from either the wild-type RT-containing p93TH253.3 or mutated RT-containing pCMV-AEB together. These spliced fragments were then ligated into pCMV-AEB.Δtat while using the BsaWI and SacI sites, yielding the six RT mutant combinations.

A VLP only expressing the PR domain of pol was generated by introducing a premature stop codon across all three reading frames at the end of the PR domain by overlap-extension PCR (using pCMV-AEB as the template and overlapping primers Odp.3227 & Odp.3228), and then ligating the spliced fragment into pCMV-AEB using the ApaI and HindIII sites to produce pCMV-AEB.ΔRT. The BsaWI/EcoRI fragment from pCMV-AEB.ΔRT was then used to replace the same fragment in pCMV-AEB.NC to produce the plasmid pCMV-AEB.NCΔRT. An Env-deficient version of pCMV-AEB.NCΔRT (pCMV-AEΔB.NCΔRT) was created by replacing the EcoRI/BlpI fragment with the same fragment from pHIS-HIV-B [55].

A mammalian-adapted Escherichia coli β-lactamase-HIV-1 Vpr fusion protein (BlaM-Vpr) expression plasmid encoding an AE clade Vpr (pMM93TH) was generated by PCR amplification of the vpr from p93TH253.3 introducing the flanking BamHI and XhoI sites (Odp.3446 & Odp.3447). These sites were then used to ligate the fragment into pMM310 (obtained from NIH ARP) [56] after the removal of the insert.

### 2.5. Production of Viral Particles

HEK 293T cells were transfected with one or more DNA plasmids while using Lipofectamine^®^ 2000 transfection reagent (Thermo Fisher Scientific), according to the manufacturer’s guidelines using approximately 250ng DNA per cm^2^ of flask or plate surface area. The transfected cells were cultured for 48–72 h in Opti-MEM media (Thermo Fisher Scientific) before harvesting culture supernatant. The supernatant was clarified by centrifugation (1200× *g*/20 min/4 °C) and 0.45 μm filtration before the particles were pelleted by overlaying supernatant on a cushion of 20% (*w/v*) sucrose in TNE buffer (10 mM Tris-HCl [pH 8.0], 1 mM EDTA, 150 mM NaCl) in Ultra-Clear™ Thinwall Tubes (Beckman Coulter, CA, USA) and centrifuged at 100,000× *g*/2 h/4 °C in a swinging-bucket rotor. Once the particles were pelleted, the supernatant and cushion were poured off and the tube was allowed to drain inverted for 1–2 min. before PBS was added to the pellet. Viral particle pellets were allowed to soften on ice with gentle agitation before the particles were resuspended and aliquoted before being snap-frozen in liquid nitrogen and stored at −80 °C before use.

### 2.6. Rate Velocity Centrifugation

Linear 6–18% (*w/v*) iodixanol gradients were prepared in 13.2 mL Ultra-Clear™ Thinwall Tubes approximately 24 h before use by gently layering four different iodixanol solutions (6, 10, 14, and 18% (*w/v*) made up in PBS), from densest to lightest using 2.8 mL per layer and allowing the layers to diffuse at 4 °C. The viral particles were concentrated, as described above, except the sucrose/TNE cushion was substituted with 5% (*w/v*) iodixanol in PBS. Viral particle pellets (resuspended in approximately 500 μL PBS) were overlaid onto the iodixanol gradient and centrifuged at 238,000× *g*/95 min/4 °C (without braking) in a swing-bucket rotor. Gradient fractions were collected from the top of the tube beginning with the uppermost, ~500 μL iodixanol-free fraction of buffer created upon the application of the sample to the gradient and then 12~930 μL fractions of increasing density. The fractions were stored at −80 °C until subsequent analysis. For SDS-PAGE analysis, the fractions were concentrated by TCA precipitation prior to loading on gels.

### 2.7. Transmission Electron Microscopy

The HEK 293T cells were transfected, as described above, with virus or VLP expression plasmids, or the previously described negative control plasmid pCMV-EGFP with the exception that cells were cultured in growth medium following transfection. Forty-eight hours after transfection, the growth medium was removed and cells were fixed with 3% (*w/v*) glutaraldehyde in PBS at 4 °C overnight. The cells were washed 2–3 times with cacodylate buffer (0.1M sodium cacodylate, pH 7.4), followed by fixation in 1% (*w/v*) OsO4 in cacodylate buffer for 40 min. at room temperature (RT). Following washing with cacodylate buffer, the samples were dehydrated in graded acetones and subsequently infiltrated with Procure resin (ProSciTech, QLD, Australia) and polymerized at 65 °C for 24–72 h. An Ultramicrotome EM UC7 (Leica, Wetzlar, Germany) that was equipped with a Diatome diamond knife was used to cut 70 nm sections that were collected on formvar/carbon-coated copper mesh grids (ProSciTech). Sections were imaged at 120kV using a Tecnai™ G2 Spirit (FEI, OR, USA) equipped with a CCD camera (Gatan, CA, USA). The embedding, sectioning, and imaging of samples were performed at the Melbourne Advanced Microscope Facility, VIC, Australia.

### 2.8. Western Blotting

Samples were electrophoresed by SDS-PAGE under reducing conditions using Tris-Glycine-buffered linear, gradient gels before being transferred to PVDF membranes (PerkinElmer, MA, USA) by wet transfer. Membranes were washed with PBS containing 0.1% (*v/v*) Tween-20 (PBST) and blocked with 5% (*w/v*) skim milk powder in PBST (Block Solution). Membranes were stained with unlabeled, primary antibodies or sera diluted in Block Solution as indicated for 3 h at RT or overnight at 4 °C. Membranes were then incubated with HRP-conjugated secondary antibody (Goat anti-Mouse IgG (H + L) or Rabbit anti-Sheep IgG (H + L) as appropriate for the primary antibody/serum host species) (Thermo Fisher Scientific) diluted 1:10,000 in Block Solution for 1 h at RT. All steps were performed with gentle agitation and PBST washes were performed after each step. Bands were visualized with SuperSignal^®^ West Pico Chemiluminescent Substrate (Thermo Fisher Scientific) and imaged with a MF-ChemiBIS 3.2 system (DNR Bio-Imaging Systems, Neve Yamin, Israel). Densitometric analysis was performed using ImageJ software (v1.51) [57].

### 2.9. Enzyme-Linked Immunosorbent Assays (ELISAs)

#### 2.9.1. p24 ELISA

MaxiSorp™ flat-bottom plates (Thermo Fisher Scientific) were coated with 3 μg/mL D7320 in 20 mM Tris-HCl (pH 8.8), 100 mM NaCl overnight at 4 °C. Plates were blocked with 300 μL/well of 5% (*w/v*) skim milk in PBS (Block Buffer) for 1 h at RT. Viral particle preparations were lysed with 1% (*v/v*) Triton™ X-100 at 37 °C for 1 h. Lysed viral particles and the p24 standards were diluted with PBS containing 0.1% (*v/v*) Triton™ X-100 and 1% (*v/v*) FBS before being added to wells and then incubated for 3 h at RT. Mouse anti-p24 mAb BC1071 was diluted to 0.5 μg/mL with Block Buffer and incubated for 2 h at RT. HRP-conjugated Goat Anti-Mouse IgG (H + L) secondary antibody diluted 1:3000 in Block Buffer supplemented with 1% (*v/v*) non-immune sheep serum (kindly provided by Carol A. Hartley, The University of Melbourne) and 0.05% (*v/v*) Tween-20 was prepared 30 min before use and then incubated in the ELISA plate for 1 h at RT. Colorimetric detection was performed with 0.1 mg/mL 3,3′,5,5′-tetramethylbenzidine (TMB) in phosphate-citrate buffer (pH 5.0) containing 2 mM H2 O2 and was incubated for 30 min. The reaction was stopped with 50 μL/well of 1 M H2 SO4, and plates were read at 450 nm. Plates were washed 4 times with PBS between each step, except following the TMB incubation and the addition of H2 SO4. All volumes were 100 μL/well unless otherwise stated.

#### 2.9.2. VLP ELISA

MaxiSorp™ flat-bottom plates were coated with 40 µL/well of VLPs or gp120 diluted in PBS overnight at RT. Plates were wash four times with PBS before being blocked with 200 μL/well of Block Buffer for 1 h at 37 °C and then washed two times with PBS. Env-specific human mAbs were diluted in Block Buffer and 40 μL/well incubated for 2 h at 37 °C before washing the plates four times with PIE (PBS containing 2 mM imidazole and 1 mM EDTA). HRP-conjugated Goat Anti-Human IgG (H+L) secondary antibody (Thermo Fisher Scientific) diluted 1:2000 in Block Buffer was incubated with 50 μL/well for 1 h at RT before washing plates with PIE six times. Colorimetric detection was then performed, as described above. The trendlines were fitted to average optical density values while using GraphPad Prism version 7.04 (GraphPad Software, La Jolla, CA, USA).

### 2.10. Reverse Transcriptase Assay

The assay was based on previously described protocols [58,59]. Briefly, each reaction mixture contained 10 μL of neat or diluted viral supernatant, or iodixanol density gradient fraction in a 40 μL volume with 50 mM Tris-HCl (pH 7.8), 7.5 mM KCl, 5 mM MgCl2, 2 mM DTT, 0.1% (*w/v*) NP-40, 5 μg/mL polyadenylic acid, 250 ng/mL oligo dT_12–18_, and 7.5 μCi/mL [α-52P]dTTP (PerkinElmer). The reaction was incubated at 37 °C for 2 h before 10 μL of each reaction was spotted in duplicate onto DE81 DEAE cellulose filter paper (GE Healthcare Life Sciences, Little Chalfont, UK). The filter paper was washed with saline-sodium citrate buffer and then ethanol, before being air-dried and exposed to a BAS-MS-IP 2340 imaging plate and read on a Molecular Imager FX™ Pro (Bio-Rad, CA, USA) and then analysed with Quantity One^®^ software (Bio-Rad).

### 2.11. Virus Fusion Assay

Viral particles that were used for the fusion assay were produced, as described above, with the exception that cells were co-transfected with pMM93TH at one-fifth the mass of the total DNA. The viral fusion assay was based on previously published protocols [56]. Briefly, using 96-well flat-bottom plates, 5 × 10^5^ CEM.NK^R^ CCR5+ cells were added to wells that were loaded with viral particles containing BlaM-Vpr (ranging between 0.01–1000 ng p24, as determined by p24 ELISA) diluted in Opti-MEM (final total volume 200 μL). The samples of immature VLPs, for which the p24 concentration could not be accurately quantified by the p24 ELISA, were loaded according to their gp120 concentration and normalized to the highest gp120-containing sample. Spinoculation of the cells and viral particles was performed at 800×g for 1 h at 18–22 °C, and then incubation for 2 h at 37 °C, 5% (*v/v*) CO2 to ensure fusion reached completion. The media containing excess viral particles was removed from the cells and the cells were washed with CO2 Independent Medium (Thermo Fisher Scientific) and loaded with CCF2/AM dye from the LiveBLAzer™ FRET-B/G Loading Kit (Thermo Fisher Scientific), according to the manufacturer’s instructions. Briefly, the loading solution contained 2 μM CCF2/AM, 139.2 μM acetic acid, 0.8 mg/mL Pluronic-F127R in CO2 Independent Medium, and the cells were incubated in 100 μL loading solution per well for 1 h at RT. Cells were washed twice with CO2 Independent Medium and then incubated for 14–16 h at 18–22 °C in 200 μL/well CO2 Independent Medium that was supplemented with 10% (*v/v*) FBS and 2.5 mM probenecid. The cells were then analysed by flow cytometry using a BD LSRFortessa™ (The University of Melbourne Flow Cytometry Core Platform, VIC, Australia) and endpoint BlaM-mediated CCF2/AM dye cleavage (% fusion) was assessed by measuring the shift in emission fluorescence detected at 525 ± 50 nm (uncleaved) to 450 ± 40 nm (cleaved) when excited by a 405 nm laser. The p24 concentration yielding 50% fusion was interpolated from three-parameter logistic curves that were fitted using GraphPad Prism v7 software.

## 3. Results

### 3.1. HIV-1 AEB VLPs Are Mostly Immature and Irregularly Sized

**Figure 1:**
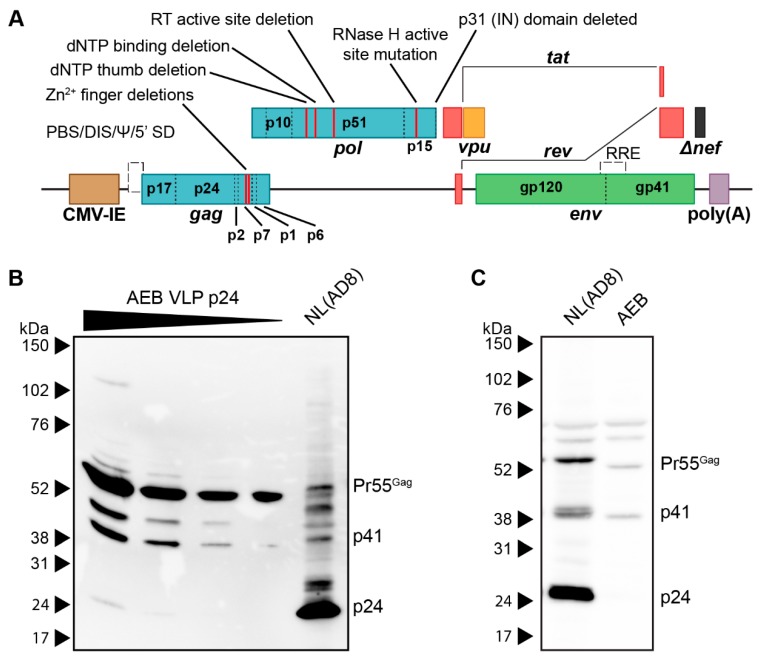
Genetic organisation and Gag processing in AEB VLPs. (**A**) Schematic representation of the AEB VLP expression cassette. Red lines within *gag* and *pol* indicate the locations of deletions and mutations, and the specific mutation is described by the indicated text. CMV-IE, cytomegalovirus immediate early promoter; poly(A), polyadenylation signal; PBS, primer binding site; DIS, dimer initiation site; Ψ, retroviral Psi packaging element; 5′ SD, 5′ splice donor; RRE, Rev responsive element; RT, reverse transcriptase; IN, integrase. (**B**) Representative Western blot using anti-p24 (#24-4) on NL(AD8) virus and 2-fold increments of AEB VLPs. (**C**) Representative Western blot using anti-p24 (#24-4) on equal volumes of cell lysate (from cell number-equalized samples) from cells expressing NL(AD8) and AEB VLPs. For both (**B**) and (**C**), 8–16% SDS-PAGE was used to resolve samples. Positions of Gag proteins are indicated on the right. Protein sizes were indicated by Amersham ECL Full-Range Rainbow Molecular Weight Markers (GE Healthcare Life Sciences) and are shown on the left.

The AEB VLP expression cassette, which encoded an AE clade *gag* and *pol* with a B clade *env*, was previously utilized as a DNA vaccine vector [37]. In this vector (Figure 1A), safety was maximized by multiple mutations and deletions within the *gag*, *pol*, and *nef* ORF to abrogate enzymatic activity, such as RT function, RNA encapsidation, and IN activity. Specifically, the NC zinc (Zn^2+^) fingers were deleted by removing residues C15–C18 and C36–C39 (HXB2 numbering used here and throughout this manuscript); the RT was inactivated by the deletion of dNTP thumb (K65–L74), dNTP binding (D113–Y115), and active site (Y183–D186) residues; the RNase H active site was mutated (E38Q); the entire IN domain was deleted; a frameshift mutation was introduced at D36 of Nef, which created a premature stop codon at position 37. The *vpr* and *vif* genes and both 5′ and 3′ LTR promoters were completely removed, while *vpu*, *env*, *tat*, and *rev* were left intact. Replication-competent NL(AD8) (NL4-3 virus encoding an AD8 strain *env*) and increasing volumes of AEB VLP preparations were compared by anti-p24 Western blotting to assess the viral composition of these VLPs,. At the lowest VLP loading, only unprocessed (Pr55^Gag^) Gag was detected with increased loading revealing an additional matrix-capsid partial Gag processing fragment (p41) and another approximately 45 kDa partially processed fragment (Figure 1B). At the highest VLP loading, a further faint, fully-processed p24 band was observed along with the unprocessed and partially processed Gag species. In comparison, the NL(AD8) was predominantly composed of fully-processed p24, which is consistent with the structure of mature HIV-1 virions. The cell lysate from NL(AD8)-transfected cells showed an increased amount of unprocessed Pr55^Gag^, as consistent with Gag polyproteins not undergoing proteolysis until after budding (Figure 1C). However, a large amount of p24 was also detected in the lysate, which likely derived from the recently budded virions sticking to the cell membrane. Analysis of the cell lysate from the AEB particle-producing cells showed a similar distribution of Pr55^Gag^ and p41, as seen within the cell-free VLPs (Figure 1C), suggesting little to no additional Gag cleavage occurred following particle budding. This aberrant Gag processing profile was unexpected, given the AEB VLP *pol* gene contained an unmodified PR sequence and therefore was anticipated to express functional PR enzyme.

**Figure 2:**
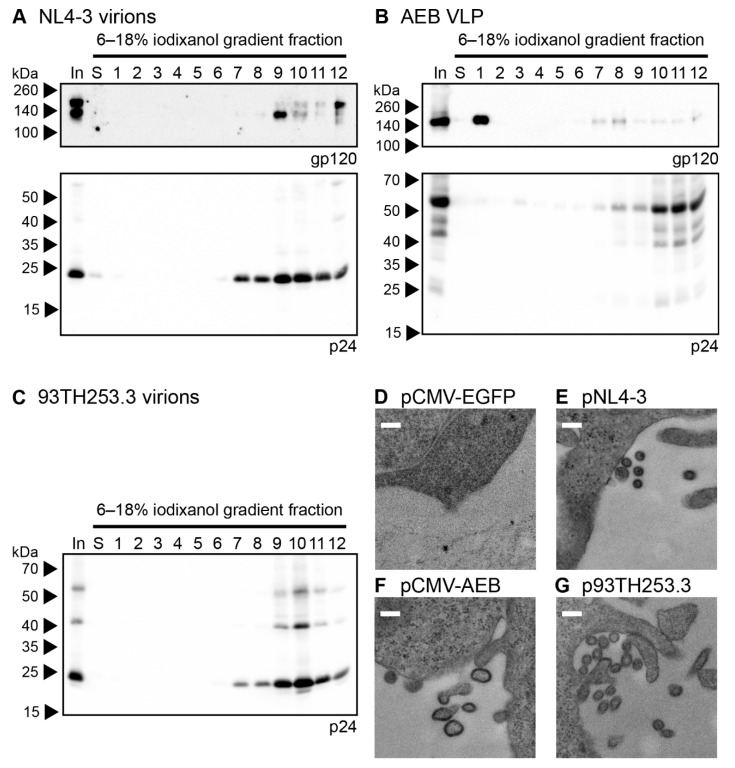
Morphology of AEB VLPs and their parental viruses. Rate velocity centrifugation of (**A**) NL4-3 virions, (**B**) AEB VLPs, and (**C**) 93TH253.3 virions was performed. A sample of the VLP concentrate prior to centrifugation (In), the uppermost iodixanol-free fraction of buffer created upon application of sample to the gradient (S), and gradient fractions of increasing density (1–12) were resolved by 6–18% SDS-PAGE before Western blotting using anti-gp120 (DV-012, upper panel) and anti-p24 (BC1071, lower panel). For all panels, protein sizes were indicated by Spectra Multicolor Broad Range Protein Ladder (Thermo Scientific) and are shown on the left. For (**A**) note the absence of an anti-gp120 upper panel due to 93TH253.3 not expressing a functional Env. Representative Western blots are shown. HEK 293T cells transfected with (**D**) pCMV-EGFP, (**E**) pNL4-3, (**F**) pCMV-AEB, and (**G**) p93TH253.3 were imaged by transmission electron microscopy (TEM). Representative images are shown for each sample. White scale bar represents 200 nm.

The morphology of the AEB VLPs was compared to infectious NL4-3 virions and AE clade virions from the 93TH253.3 isolate (from which AEB *gag* and *pol* were derived) by iodixanol density gradient rate velocity centrifugation. The samples of the initial VLP concentrate (In), the uppermost iodixanol-free fraction of buffer created upon the application of sample to the gradient (S) and gradient fractions of increasing density (1–12), were analysed by anti-gp120 and anti-p24 Western blotting. The NL4-3 and 93TH253.3 particles (as indicated by p24) mainly sedimented to fractions 9–10 (Figure 2A and C, lower panels, respectively) with incorporated Env also being detected in the same fractions for NL4-3 (Figure 2A, upper panel), which was consistent with previous reports [60,61]. Interestingly, an increased proportion of uncleaved Env (gp160) was observed in fraction 12 for the NL4-3 preparation, which may represent cellular microvesicles or aggregates bearing non-functional Env given the ratio of Env to Gag staining intensity was higher than in fractions 9–10. No anti-gp120 panel is shown for 93TH253.3, since its proviral sequence contains a premature stop codon in *env* [52]. The AEB VLP p24 staining was predominantly in fractions 10–12, demonstrating that the VLPs bore a different shape and/or size when compared to infectious virions, and they were composed of incompletely processed Gag (Figure 2B, lower panel). The weaker Gag staining in less dense fractions with concomitant Env staining may suggest variable particle morphology with associated differences in Env incorporation. The relatively large amount of Env in fraction 1 suggested that significant gp120 dissociation from the particle membrane had occurred either before or during centrifugation (Figure 2B, upper panel), which was expected given the shedding of gp120 from the surface of viral particles that has been reported previously [42,62].

VLP morphology was further assessed by transmission electron microscopy (TEM) of transiently-transfected cells, with EGFP-expressing cells being used to confirm no endogenous particle budding from the plasma membrane (Figure 2D). As expected, particles of approximately 120 nm in diameter were seen budding and associated with the plasma membrane of cells transfected with pNL4-3 and p93TH253.3 (Figure 2E,G, respectively). At the plasma membrane of cells expressing pCMV-AEB, irregularly shaped and often tube-like particles were visible with diameters varying between 100 and 200 nm (Figure 2F). This suggested that, rather than simply being proteolytically immature due to PR inactivity, the VLPs were not assembling appropriately at the plasma membrane, which was likely due to Gag/Gag-Pol polyproteins being misfolded or their interactions being disrupted by one or more of the mutations and deletions within *gag* and *pol*.

### 3.2. Restoration of RNA Stem Loop Motifs and RNA-Interacting Protein Domains Does Not Enhance Particle Assembly and Maturation

Several of the mutations within the AEB VLP *gag* and *pol* regions had potential to cause the aberrant Gag cleavage profile that was seen in the viral particles: The complete removal of the IN domain (Figure 1A), which forms the C-terminal domain of Gag-Pol polyproteins and likely forms interactions with other Gag-Pol proteins to aid in assembly and maturation [63]. The deletion of the NC Zn^2+^ fingers that normally facilitate interactions with the HIV-1 genomic RNA (gRNA) and cellular RNA act as scaffolds to tether adjacent Gag polyproteins [64,65], facilitate oligomerisation of Gag in the cytoplasm [66], and are important for the assembly of immature virions by forming nucleation sites at the plasma membrane [67,68,69]. Furthermore, RNA stem loop structures within the gRNA regulate exposure of the residues required to bind NC [70,71], specifically residues of the Unique-5′ region (U5), which pair with a region overlapping the *gag* start codon to promote gRNA dimerisation and enhance NC binding [71].

**Figure 3:**
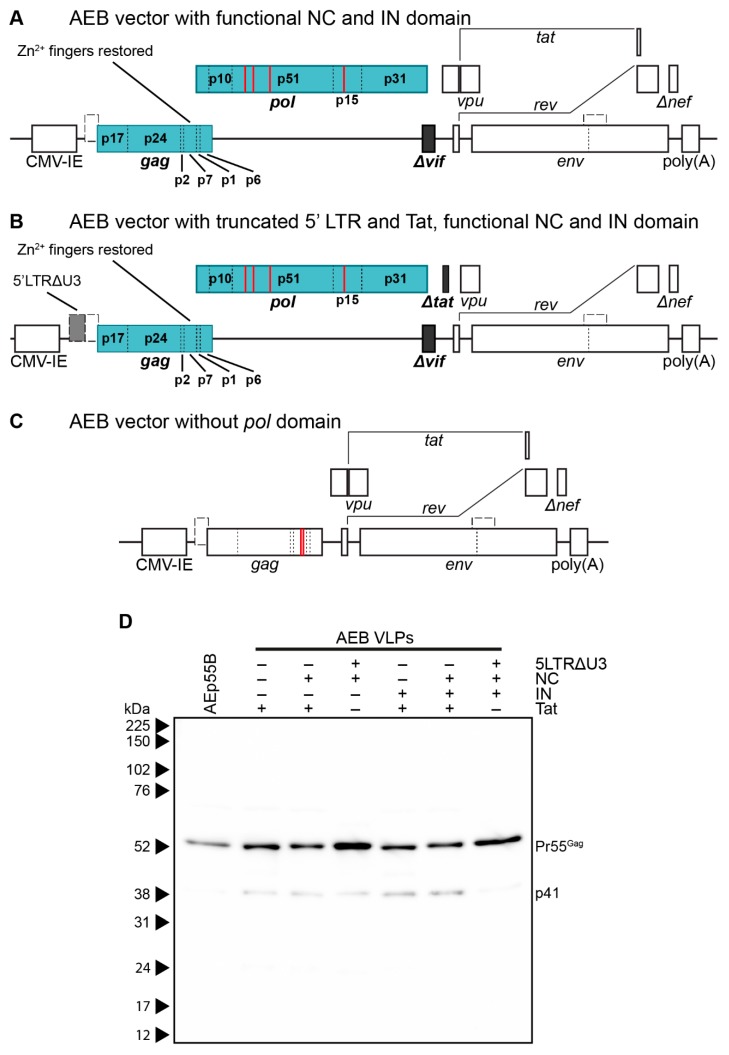
Genetic organization and characterization of VLPs expressing functional RNA binding domains and RNA packaging motifs. (**A**) Schematic representation of the pCMV-AEB.NC.IN expression cassette. (**B**) Schematic representation of the pCMV-5 LTRΔU3-AEB.NC.IN.Δtat expression cassette. (**A**) and (**B**) also represent the plasmids pCMV-AEB.NC and pCMV-5 LTRΔU3-AEB.NC, respectively, except the IN domain (p31) was absent in these plasmids. (**C**) Schematic representation of expression cassette in pCMV-AEp55 B, that lacked the entire *pol* domain. Abbreviations, deletions and modifications are as described in Figure 1A. Newly introduced or modified ORFs relative to Figure 1A are indicated by filled in blocks. (**D**) Representative Western blot following 8–16% SDS-PAGE using anti-p24 (#24-4) on purified AE clade VLPs. Unmodified AEB VLPs and VLPs from modified plasmids expressing one or more of a truncated 5′ LTR (5 LTRΔU3), functional nucleocapsid (NC), functional integrase domain (IN), and a functional Tat protein (Tat) are shown. Also shown are Gag-only VLPs (AEp55 B). The positions of Gag polyprotein cleavage products are indicated on the right and protein sizes were indicated by Amersham ECL Full-Range Rainbow Molecular Weight Markers and are shown on the left.

To assess the contribution of the RNA stem loop and RNA-interacting protein domain mutations to AEB VLP morphology, particles that were expressed from vectors with a functional NC domain (Figure 3A) or a truncated 5′ LTR and Tat with a functional NC domain (Figure 3B), both with or without an intact IN subunit, were compared by anti-p24 Western blotting. It should be noted that Tat was truncated in VLP vectors containing a truncated 5′ LTR to inhibit the interaction with the TAR stem loop that might otherwise alter RNA PolII processivity [72]. VLPs that were expressed from an AEB VLP vector lacking a functional *pol* domain (AEp55B, Figure 3C) were also included as a control for completely unprocessed Gag. As expected, the AEp55B VLPs displayed an immature profile with only a Pr55^Gag^ band observed (Figure 3D). The presence of NC Zn^2+^ fingers (labelled as NC +) with or without an IN domain (IN +/−) did not alter the Gag cleavage efficiency of AEB VLPs, with fairly consistent Pr55^Gag^ expression and no p24 being detected. When these modifications were coupled with the introduction of RNA motifs for efficient RNA scaffolding and packaging located in the truncated 5′ LTR (5LTRΔU3 +), the VLPs failed to demonstrate a mature particle phenotype, with similar proportions of Pr55^Gag^ and p41 being observed for the unmodified AEB VLPs (labelled as + for functional Tat and–for all other modifications).

### 3.3. Amino Acid Deletions within the RT Domain Block Particle Assembly and Maturation

**Figure 4:**
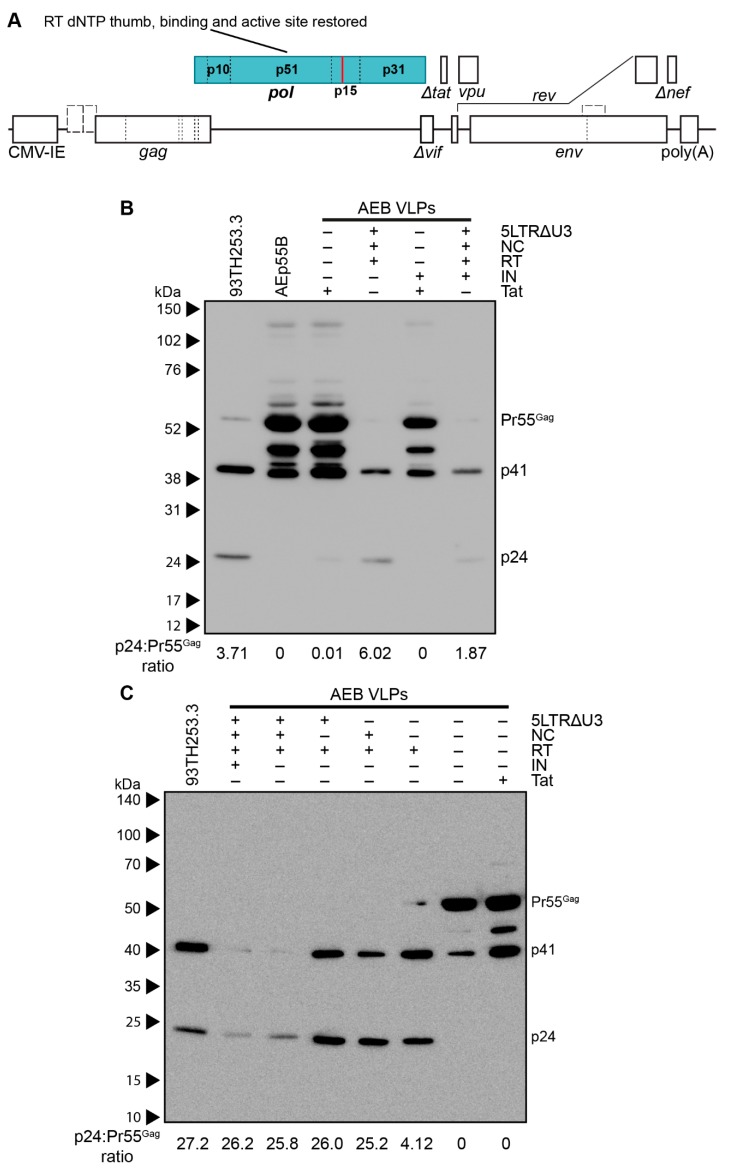
Characterization of VLPs with restored RT domain. (**A**) Schematic representation of the pCMV-5LTRΔ3-AEB.NC.RT.IN.Δtat VLP expression cassette. The VLP expression cassette of pCMV-5LTRΔ3-AEB.NC.RT.Δtat is also represented by this schematic, except that it lacks the IN domain (p31). Abbreviations, deletions and modifications are as described in Figure 1A. Newly introduced or modified ORFs relative to Figure 3B are indicated by filled in blocks. (**B**,**C**) Representative Western blots using anti-p24 (#24-4) on VLPs and the 93TH253.3 parental virus. AEB VLPs are shown along with VLPs expressing one or more of a truncated 5′ LTR (5LTRΔU3), functional nucleocapsid (NC), functional reverse transcriptase (RT), functional integrase (IN), and functional Tat protein (Tat), as indicated. All of the samples were resolved by 8–16% SDS-PAGE. The positions of Gag polyprotein cleavage products are indicated on the right and the protein sizes are indicated on the left by (**B**) Amersham ECL Full-Range Rainbow Molecular Weight Markers or (**C**) Spectra Multicolor Broad Range Protein Ladder.

The other major set of safety mutations within the *gag* and *pol* domains were those affecting RT. It has been reported that certain mutations within RT were designed to affect p51/p15 heterodimer stabilisation often decreased the infectivity of the resulting virions due to premature PR activity producing immature virions with irregular size and morphology [73]. Although the specific RT mutations that were previously reported (L234D or W239A) differed to the deletions employed in the VLP plasmids used here (deletion of K65–L74, D113–Y115, and Y183–D186), the effect of restoring a functional RT domain was assessed in the AEB VLP vectors containing functional NC and the truncated 5′ LTR with or without a restored IN domain (Figure 4A shows IN functional version). Anti-p24 Western blotting demonstrated the AEB VLPs with the 5LTRΔU3 and Tat, and the functional NC and RT domains had increased p24 to Pr55^Gag^ ratios (as measured by band densitometry) and a Gag cleavage profile that was similar to the parental 93TH253.3 virus (Figure 4B). This was a significant departure to the incomplete Gag cleavage profile that was observed for the unmodified AEB VLP (labelled as + for functional Tat and − for all other modifications), which bore greater similarity to Gag-only AEp55B VLP with p24 to Pr55^Gag^ ratios that are close to zero. The RT domain-modified VLPs with the IN domain (labelled as − for functional Tat and + for all other modifications) also demonstrated enhanced Gag cleavage relative to particles containing the IN domain, but lacking the LTR, NC, and RT domain modifications.

The role of the 5′ LTR U5 and NC Zn^2+^ fingers for proper particle assembly and maturation was assessed with VLP vectors expressing functional RT alone and in combination with the U5 or functional NC. Anti-p24 Western blotting showed that increased Gag processing was strongly associated with the presence of functional RT (RT +) with a similar Gag cleavage profile to the parental 93TH253.3 virus, regardless of other modifications in the VLP vector (Figure 4C). The VLPs that were expressed from cassettes containing both the truncated 5′ LTR and NC had relatively reduced expression of particles and the inclusion of a functional NC along with the restored RT domain slightly enhanced Gag processing, as demonstrated by an absence of Pr55^Gag^ staining, despite similar p24 levels to VLPs without the Zn^2+^ fingers present. The Tat truncation that was used in conjunction with the 5′ LTR U5 modification was also assessed in isolation (labelled as − for all modifications) to confirm that the Tat modification was not altering Gag processivity, and it demonstrated a similar Gag cleavage profile to the unmodified AEB VLP. Overall, the three RT domain deletions in the AEB VLPs appeared to be the specific cause of the aberrant Gag cleavage profile. Combinations of the three RT domain deletions (dNTP thumb, dNTP binding site, and RT active site deletions) were subsequently investigated for their contribution to the aberrant Gag cleavage profile. Analysis by anti-p24 Western blotting showed that AEB VLPs containing any one or more of the RT deletions produced particles with a similarly deficient Gag cleavage profile to that of VLPs with all three RT mutations (Supplementary Figure S1).

**Figure 5:**
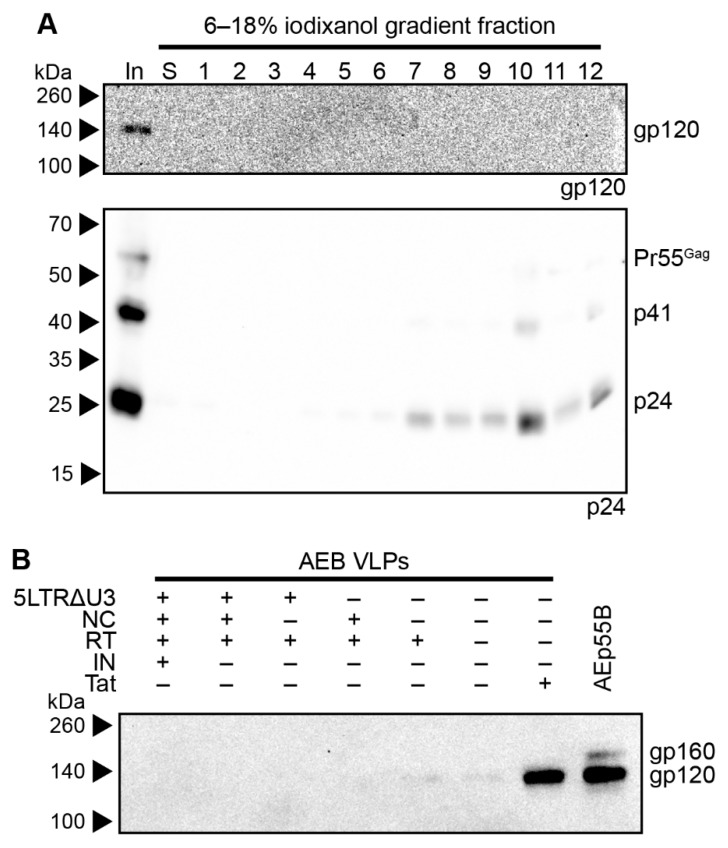
Rate velocity centrifugation of AEB.RT.Δtat VLPs and Env expression of modified AE clade VLPs. (**A**) AEB.RT.Δtat VLPs were subjected to rate velocity centrifugation. A sample of the concentrated particles prior to centrifugation (In), the uppermost iodixanol-free fraction of buffer created upon application of sample to the gradient (S), and gradient fractions of increasing density (1–12) were collected and resolved by 6–18% SDS-PAGE before Western blotting using anti-gp120 (DV-012, upper panel) and anti-p24 (BC1071, lower panel). (**B**) Gag-only AEB VLPs (AEp55B) and AEB VLPs with various modifications—a truncated 5′ LTR (5LTRΔU3), functional nucleocapsid (NC), functional reverse transcriptase (RT), functional integrase domain (IN), and functional Tat protein (Tat)—were resolved by 8% SDS-PAGE before Western blotting with anti-gp120 (DV-012). For (**A**) and (**B**), the positions of Env and the Gag polyprotein cleavage products are indicated on the right and protein sizes were indicated on the left by Spectra Multicolor Broad Range Protein Ladder.

The AEB VLPs encoding a functional RT domain and non-functional Tat (AEB.RT.Δtat) were further investigated, given that they expressed a good yield of mostly mature particles without including a functional NC domain, which is advantageous from a safety perspective. The morphology of AEB.RT.Δtat VLPs was assessed by rate velocity centrifugation through an iodixanol density gradient with samples being collected, as described previously. Anti-p24 Western blotting showed that the particles were predominantly found in fraction 10 (Figure 5A, lower panel), which was similar to the parental 93TH253.3 sedimentation profile (Figure 2C). This suggested that the shape and size of the AEB.RT.Δtat VLPs was more similar to infectious virions than the original AEB VLPs. However, it should be noted that 12 × 930 μL fractions analysed only allow for the course comparison of particle morphology. The density gradient-separated AEB.RT.Δtat VLPs also appeared to contain more Gag that was fully processed to p24 (Figure 5A) as compared to AEB.RT.Δtat VLPs that had not been fractionated (Figure 4C). Given that the rate velocity centrifugation fractions underwent TCA precipitation prior to Western blotting, this may be due to p24 being more efficiently precipitated than other Gag cleavage species, such as p41. Western blotting for gp120 revealed very low amounts of Env in the input sample and no detectable Env in the gradient fractions (Figure 5A, upper panel), despite being previously able to readily detect Env in other VLP and HIV-1 virion fractions (Figure 2B, upper panel). Anti-gp120 Western blotting on preparations of the AEp55B VLP, the original AEB VLPs, and those with combinations of the truncated 5′ LTR, functional NC, functional RT, and truncated Tat demonstrated that VLPs with an unmodified Tat reading frame (the original AEB and AEp55B VLPs) showed strong Env expression, while those encoding the “Δtat” mutation had low to no Env expression (Figure 5B).

**Figure 6:**
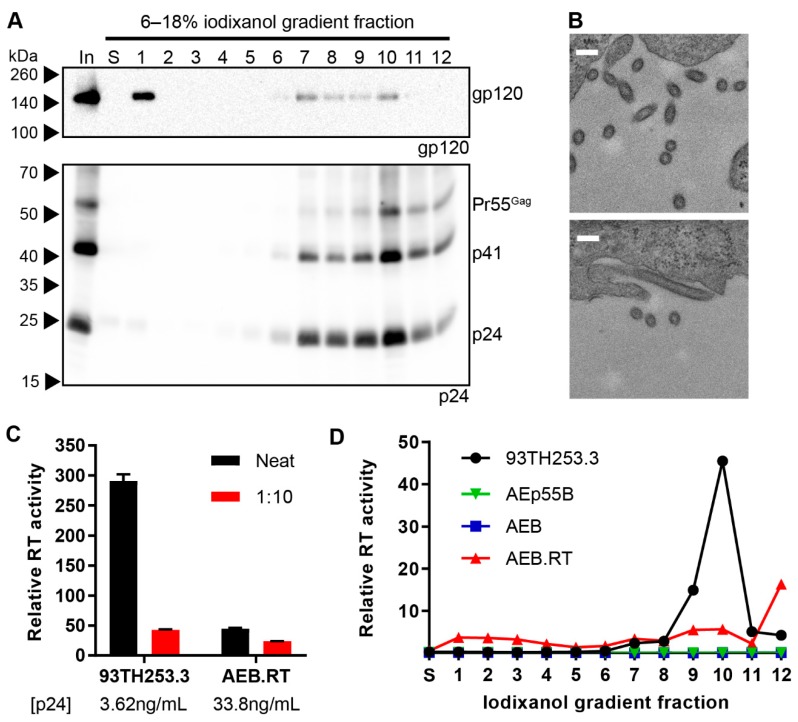
Morphology and RT activity of AEB.RT VLPs. (**A**) AEB.RT VLPs were subjected to rate velocity centrifugation. A sample of the concentrated particles prior to centrifugation (In), the uppermost iodixanol-free fraction of buffer created upon application of sample to the gradient (S), and gradient fractions of increasing density (1–12) were resolved by 6–18% SDS-PAGE before Western blotting using anti-gp120 (DV-012, upper panel) and anti-p24 (BC1071, lower panel). The positions of Env and the Gag polyprotein cleavage products are indicated on the right and protein sizes were indicated on the left by Spectra Multicolor Broad Range Protein Ladder. (**B**) Representative images of cells transfected with pCMV-AEB.RT imaged by TEM. Scale bar represents 200 nm. (**C**) The relative RT activity of neat and 1:10 diluted 93TH253.3 virions and AEB.RT VLPs as quantified by a radioactive RT activity assay. The p24 concentration of 93TH253.3 virions and AEB.RT VLPs was determined by p24 ELISA. Values represent the mean and SEM from two independent measurements. (**D**) Relative RT activity was measured in fractions S and 1–12 from rate velocity centrifugation fractions from 93TH253.3, AEp55B, AEB, and AEB.RT particle preparations. Values represent the mean of duplicate measurements.

Given that the truncation of Tat was introduced to prevent potential alterations to VLP mRNA transcription via interaction between Tat and the TAR RNA stem loop present within the truncated 5′ LTR sequence, and the truncated 5′ LTR sequence was dispensable for the efficient expression of mature-form AEB VLPs with a functional RT domain, a modified AEB.RT.Δtat VLP vector with a functional Tat ORF (AEB.RT) was investigated. These AEB.RT VLPs showed increased levels of Env expression and demonstrated a similar particle size and/or shape as AEB.RT.Δtat VLPs by rate velocity centrifugation (Figure 6A). Env co-localisation with p24-containing fractions was consistent with Env incorporation into the particles. It is worth noting there was a significant amount of Env in fraction 1 most likely indicating soluble gp120 that had been shed from the VLPs before or during sedimentation, which is an expected phenomenon that was previously noted for AEB VLPs. The morphology of the AEB.RT VLPs was further assessed by TEM of the pCMV-AEB.RT plasmid-transfected cell sections (Figure 6B). The AEB.RT particles were similarly sized to virions budding from cells that were transfected with p93TH253.3 (Figure 2G); however, the VLPs displayed a distribution of circular and oval shapes (Figure 6B) that was distinctly different to the infectious virions. It should be noted that these two shapes might have resulted from a single population of ovaloid particles that are visualized from cuts along their short and long axes. The AEB.RT VLPs were approximately 100–120 nm in diameter across the short axis and up to approximately 200 nm across the long axis for those with an oval shape. Overall, these VLPs were more consistently shaped than what was previously observed for AEB VLPs (Figure 2F). The restored RT domain in AEB.RT VLPs also resulted in these particles containing a functional RT enzyme that had measurable activity in the RT assays (Figure 6C). However, the VLPs demonstrated approximately 60-fold lower activity relative to 93TH253.3 virions when normalized by p24 concentration. Analysis of the iodixanol density gradient rate velocity centrifugation fractions showed that the RT activity of AEB.RT VLPs was present in the p24-containing fractions, but there was a significant increase in the amount of activity in the densest fraction (fraction 12) that did not correlate with an increase in p24 protein (Figure 6A,D). This was likely due to aggregated or cellular microvesicles, which contained a functional RT enzyme, which rapidly sedimented during centrifugation and appeared at the bottom of the gradient.

### 3.4. Removal of the Entire RT Domain Facilitates Expression of VLPs Resembling Mature Virions

**Figure 7:**
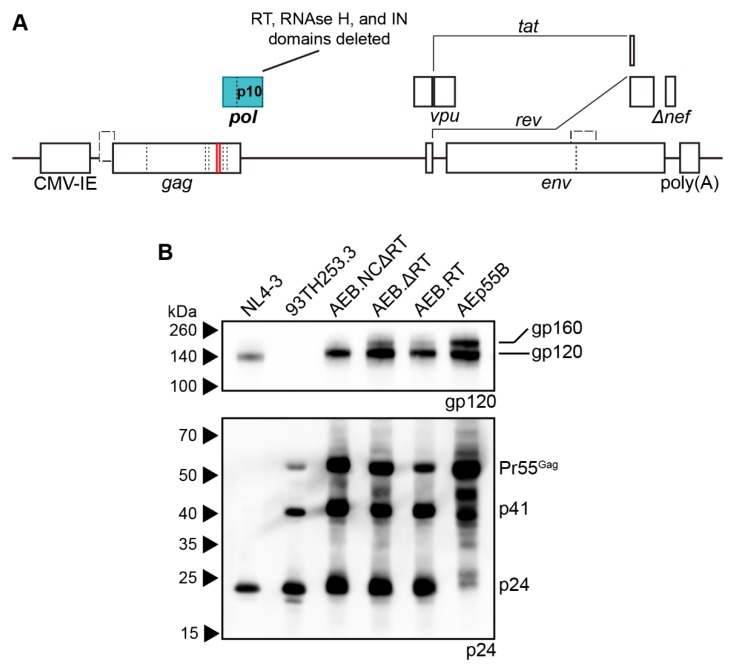
Genetic organization and Gag processing of AEB VLPs lacking RT domains. (**A**) Schematic representation of the pCMV-AEB.ΔRT and pCMV-AEB.NCΔRT expression cassettes, except for the absence of Zn^2+^ finger deletions for the latter. Abbreviations, deletions and modifications are as described in Figure 1A. Newly introduced or modified ORFs relative to Figure 1A are indicated by filled in blocks. (**B**) Representative Western blot using anti-gp120 (DV-012, upper panel) and anti-p24 (BC1071, lower panel) on NL4-3 and 93TH253.3 virions, AEB VLPs with deleted RT and RNase H domains (AEB.ΔRT) and those with an additional functional NC (AEB.NCΔRT). AEB.RT VLPs (functional RT domain) and Gag-only VLPs (AEp55B) are also shown. The samples were resolved by 8–16% SDS-PAGE. The position of Env and the Gag polyprotein cleavage products are indicated on the right and protein sizes were indicated on the left by Spectra Multicolor Broad Range Protein Ladder.

Alternative approaches to expressing mature-form VLPs without including a functional RT domain were investigated to achieve a safer VLP expression plasmid. Given that the original RT safety mutations and deletions likely destabilized the Gag-Pol polyprotein and prematurely activated the PR enzyme (based on reports of RT “primer grip” mutations inducing premature PR activity [73]), whether complete inactivation of the RT and RNase H domain (ΔRT) could circumvent this problem was assessed. AEB VLPs with a *pol* truncation C-terminal to the PR domain (AEB.ΔRT, genetic representation, as shown in Figure 7A) were generated along with an identical vector that additionally contained a functional NC domain (AEB.NCΔRT), given that less uncleaved Gag was observed in AEB VLPs with both a functional NC and RT domain when compared to a functional RT domain alone (Figure 4C). Anti-p24 Western blotting demonstrated that both AEB.NCΔRT and AEB.ΔRT VLPs displayed a similar distribution of Gag cleavage products as the RT domain-containing AEB.RT VLPs and marginally less Gag processing as compared to the parental 93TH253.3 virus, despite the ablation of the RT and RNase H domains (Figure 7B, lower panel). Analysis by anti-gp120 Western blotting showed that the AEB.NCΔRT VLPs appeared to predominantly contain cleaved Env (gp120), while AEB.ΔRT VLPs incorporated a small portion of uncleaved Env (gp160) (Figure 7B, upper panel). Given the intended use of the VLPs as immunogens that, ideally, should efficiently present cleaved Env and that the NC Zn^2+^ finger deletions are known to have little effect on the already low level of RNA encapsidation in 93TH253.3 virions (Anne Ellet, The University of Melbourne, personal communication), the AEB.NCΔRT VLPs were utilized for further analysis.

**Figure 8:**
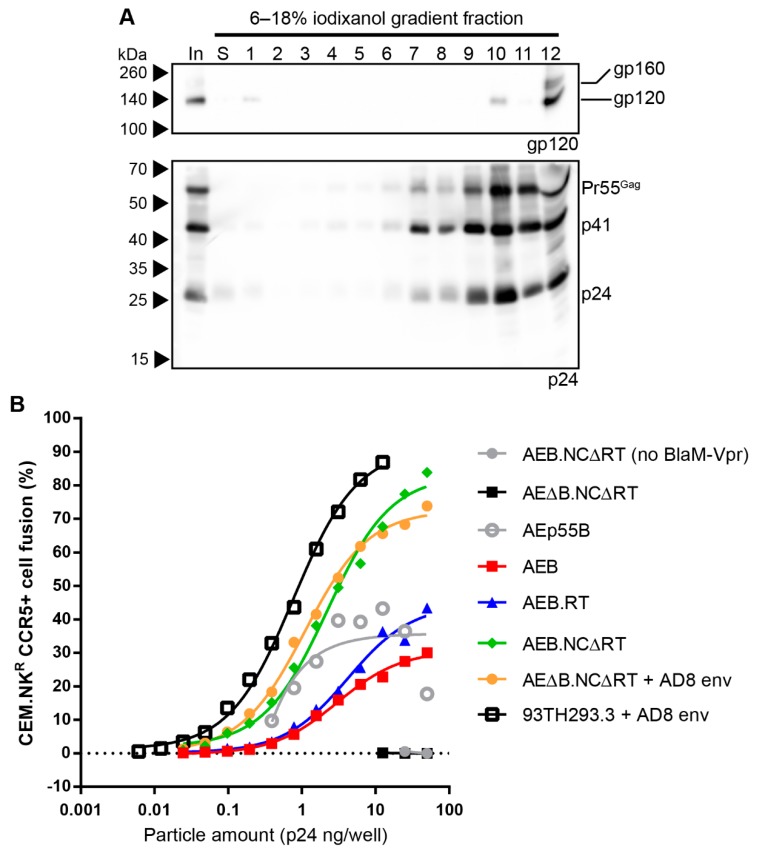
Rate velocity centrifugation and fusibility of AEB.NCΔRT VLPs. (**A**) AEB.NCΔRT VLPs were subjected to rate velocity centrifugation. A sample of the concentrated particles prior to centrifugation (In), the low-density fraction containing the particles prior to centrifugation (S), and gradient fractions of increasing density (1–12) were collected and resolved by 6–18% SDS-PAGE before Western blotting using anti-gp120 (DV-012, upper panel) and anti-p24 (BC1071, lower panel). The position of Env and the Gag polyprotein cleavage products are indicated on the right and protein sizes were indicated on the left by Spectra Multicolor Broad Range Protein Ladder. (**B**) Viral fusion assays were performed on various VLPs and the parental 93TH253.3 virus all of which were incorporating B clade Env (AD8 strain) (except for AEB.NCΔRTΔenv that lacks a functional Env). Endpoint fusion was assessed for varying amounts of particle inputs indicated, as determined by p24 ELISA. The loading of AEp55B VLPs was determined by gp120 Western blotting and was normalized to the highest gp120-containing sample loaded (AEB.RT) and plotted using the corresponding p24 concentrations (sample gp120/p24 concentration ratios detailed in Table S2). Values represent the mean of duplicate samples.

The morphology of the mature-form AEB.NCΔRT VLPs was assessed by rate velocity centrifugation, where anti-24 Western blotting showed the VLPs sedimented between fractions 7–12, with a peak in fraction 10 (Figure 8A, lower panel). This was similar to the distribution observed for their parental 93TH253.3 virions (Figure 4C), which suggested that the VLPs were approximately the same shape and/or size. Western blotting for gp120 showed Env associated with these VLPs, as indicated by a gp120 band in fraction 10, but there was also some Env detected in fraction 1, likely due to shedding from the VLPs, and gp120 and a smaller amount of gp160 within fraction 12 (the bottom fraction) (Figure 8A, upper panel). The Env within the bottom fraction was likely associated with cellular protein aggregates or microvesicles, given that there was not a concomitant increase in Gag staining with the increased Env staining.

Virus fusion assays were performed, since viral particle fusion has been reported to be inhibited by an uncleaved Gag polyprotein shell [74] and to further assess the degree of VLP maturation. The AEB.RT, AEB.NCΔRT, and unmodified AEB VLPs were compared to the parental 93TH253.3 virions along with the immature control AEp55B VLPs. 93TH253.3 virions pseudotyped with AD8 Env were generated, given that p93TH253.3 does not express a functional Env. To ensure that expressing the Env from a separate plasmid did not alter the particle fusion activity, Env-deficient AEB.NCΔRT VLPs (AEΔB.NCΔRT) were also pseudotyped with AD8 Env for comparison. As expected, non-pseudotyped AEΔB.NCΔRT VLPs showed no fusion activity and confirmed that no Env-independent fusion was possible (Figure 8B). AEp55B VLPs, despite being immature, showed low fusion activity that did not follow a typical dose-dependent logistic curve. The unmodified AEB VLPs required more than 100-fold more p24 for similar levels of fusion than the parental 93TH253.3 virions (pseudotyped with AD8 Env), and they mediated less fusion than the immature VLPs. The proteolytically mature AEB.RT VLPs mediated more fusion than AEB VLPs, but they still required a more than 10-fold greater p24 input to reach the level of fusion that is seen with the 93TH253.3 virions. The mature-form AEB.NCΔRT VLPs that were produced either by pseudotyping or from a single, Env-functional vector yielded much higher fusion activity than the other VLPs and they were only 2.7- (pseudotyped) or 3.5-fold (single vector) less efficient than the infectious 93TH253.3 virions.

### 3.5. Mature-form VLPs Can Present Anti-Env Antibody Epitopes

**Figure 9:**
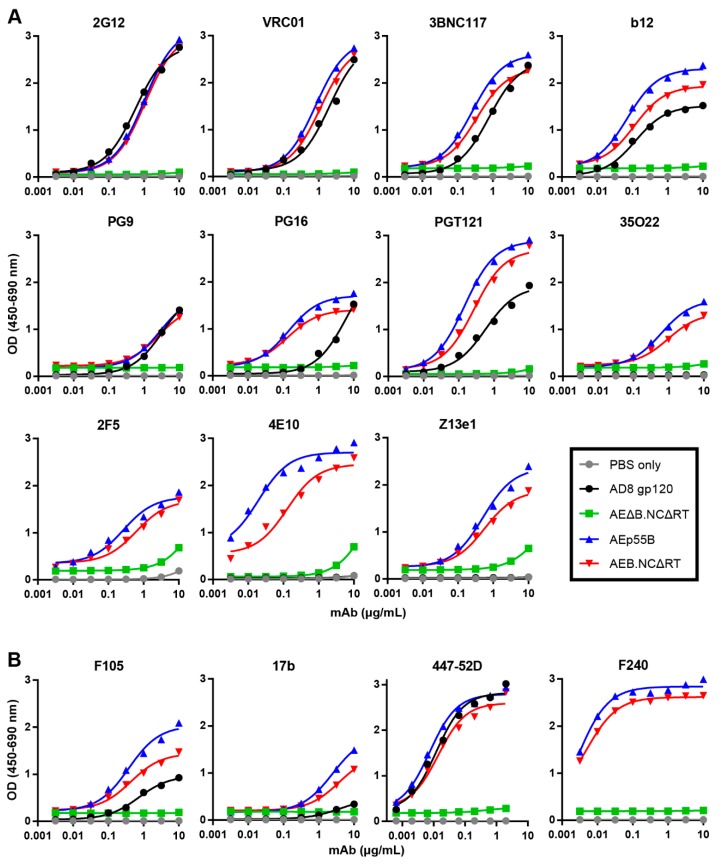
Comparison of anti-Env antibody binding to VLPs and soluble gp120. Representative ELISA binding curves of (**A**) bNAbs 2G12, VRC01, 3BNC117, b12, PG9, PG16, PGT121, 35O22, 2F5, 4E10, and Z13e1, and (**B**) poorly and non-neutralizing antibodies F105, 17b, 447-52D, and F240 to AD8 gp120, AEB.NCΔRT VLPs, AEp55B VLPs, AEΔB.NCΔRT VLPs, and a PBS only control. For both (**A**) and (**B**) VLP ELISAs were performed using concentrated AEB.NCΔRT and AEp55B VLPs equivalent to 250 ng/well purified AD8 gp120, as determined by anti-gp120 Western blotting. The loading of AEΔB.NCΔRT VLPs was equalized to the AEB.NCΔRT VLPs by p24 content, as determined by p24 ELISA.

To assess whether the extensive safety mutations in the mature-form AEB.NCΔRT VLPs prevented the incorporation and presentation of correctly folded Env, ELISAs were performed to determine the binding of bNAbs and poorly or non-neutralizing antibodies to Env-equalized amounts of AEB.NCΔRT and AEp55B VLPs. Recombinant AD8 gp120 (equalized to the VLP Env content) and Env-deficient, mature-form VLPs (AEΔB.NCΔRT) (equalized to VLP number) were included for comparison. The Env-bearing VLPs and gp120 showed comparable binding curves for 2G12 (Figure 9A), which confirmed that the loading of Env was approximately equivalent, given that 2G12 binds a carbohydrate-only epitope that is less dependent on Env conformation [75]. Overall, the Env that was presented on the AEB.NCΔRT VLPs was antigenically similar to Env incorporated into the AEp55B VLPs, although there was a trend towards marginally greater reactivity to Env on AEp55B VLPs. The VLP-associated Env recognition of the CD4bs bNAbs VRC01, 3BNC117, and b12 was comparable to soluble gp120. Both of the VLPs displayed a similar pattern of binding to bNAbs being directed to the Env V2 apex and V3 N-glycan patch regions: the VLP-associated Env bound PG16 and PGT121 more strongly than the gp120, but all displayed similarly low binding to PG9. Both VLPs were able to bind the bNAbs 35O22, 2F5, and Z13e1 with similar efficiency. In contrast, markedly higher binding to 4E10 was observed for the AEp55B VLPs when compared to AEB.NCΔRT VLPs. It should also be noted that there was also detectable non-specific interaction at the higher mAb concentrations between the Env-deficient VLP and bNAbs targeting the gp41 MPER (2F5, 4E10 and Z13e1). The binding of poorly neutralizing antibodies was relatively conserved between both VLPs (Figure 9B). Soluble gp120 was bound less strongly than when Env was presented on AEp55B and AEB.NCΔRT VLPs for the poorly neutralising gp120-specific mAbs F105 and 17b. The trend in binding between AEp55B and AEB.NCΔRT VLPs for these anti-gp120 mAbs and the anti-gp41 non-neutralising mAb F240 was similar, although the AEp55B VLPs trended towards slightly higher reactivity towards F105, 17b, and F240 as compared to AEB.NCΔRT VLPs.

## 4. Discussion

Typically, HIV-1 VLPs are expressed by co-transfecting an Env-deficient particle expression vector with a separate Env-expression plasmid. This facilitates the rapid assessment of various Env strains or it is used to express viral particles that resemble infectious virus, but are not themselves replication-competent [20,76,77]. These systems limit the use of the VLPs as immunogens for Env to in vitro-production and subsequent injection into vaccine recipients. Apart from the need to inactivate such VLPs before they can be safely used in an uninfected individual [30], the production of HIV-1-based VLPs is a laborious task that is difficult to scale-up for population-level vaccination efforts [34]. Therefore, single plasmid VLP expression vectors with sufficient safety mutations to prevent infection represent an alternative method for expression of Env immunogens. A single expression cassette is highly amenable for DNA plasmid or viral vector vaccinations, since the need for multiple plasmids to be delivered to the same site or cell in vivo is not required [78]. Such vectors are also advantageous for the in vitro-production of VLPs since the transfection efficiency of a single plasmid into a given cell will be higher than that of two or more plasmids. However, the introduction of mutations to render single-plasmid vectors safe for human-use can affect the assembly of viral structural proteins during particle formation, such that the resulting VLPs are not representative of infectious HIV-1 virions. Therefore, here we focused on the design of single plasmids that are capable of expressing VLPs that resemble mature virions while still retaining safety.

The expression of Pr55^Gag^ alone is sufficient to mediate the assembly and budding of HIV-1 VLPs from the cell membrane [79,80]; however, the resulting particles will not undergo proteolytic maturation as normally occurs for infectious HIV-1 virions without the co-packaging of the viral PR domain contained within the Pr160^Gag-Pol^ polyprotein [81,82,83]. Instead, Gag-only VLPs resemble immature viral particles that have a different morphology from mature, infectious virions in terms of internal protein arrangement often characterized by the absence of a dense conical core [84]. Additionally, maturation results in the disassembly of the rigid Gag lattice beneath the viral membrane that then facilitates Env clustering on the particle surface [26]. VLPs resembling mature virions would, therefore, facilitate the multivalent presentation of Env oligomers and likely enhance Env immunogenicity in a vaccine setting [85]. The close multimeric display of antigen, as is expected with clustered Env on a mature VLP, is an ideal geometry for optimal B cell receptor cross-linking and activation [86,87]. Additionally, this immunogen display may result in a lowering of the threshold for B cell activation and an enhancement of long-lived plasma cell differentiation [88]. VLPs resembling mature virions are also less likely to induce cellular responses to the Gag polyprotein, as suggested by observations of viral particles with a maturation defect (either by mutation or by protease-inhibitor treatment) mediating the strong cellular responses [28]. Therefore, the Env presented on mature VLPs may be the predominant focus of immune responses when used as an immunogen in a vaccine setting.

The initial single-plasmid AEB VLP expression vector yielded particles with incomplete proteolytic cleavage of Gag, as demonstrated by comparatively more Pr55^Gag^ than p24 in particles pelleted from cell supernatant, which was indicative of immature HIV-1 virion formation, despite the VLP expression vector encoding *pol* with a functional PR sequence. The partial Gag polyprotein cleavage products that were observed by anti-p24 Western blotting (p41 and a ~45 kDa capsid-containing fragment) indicated that the viral PR was at least partly functional. The functional PR normally exists as a dimer following autocatalytic cleavage and release from the multimerized Pr160^Gag-Pol^ polyproteins, which forms via interactions between the N- and C-terminal regions flanking the precursor PR domain [89,90]. For the VLP expression vector, the *pol* sequence contained a significant truncation via removal of the IN domain. However, the restoration of the IN domain in combination with functional NC or RNA stem loop structures did not alter the immature VLP profile, which suggested that inefficient Pr160^Gag-Pol^ multimer formation and PR activation were not responsible. Furthermore, rate velocity centrifugation showed that the AEB VLPs more rapidly sedimented than infectious 93TH253.3 virions expressed from the proviral sequences that the VLP vector was derived from. This showed that the size and/or shape of the VLPs was distinct from normal HIV-1 virions, which indicated a defect or difference in the assembly of the VLPs due to one or more of the mutations within *gag* and *pol* to remove their replicative functions. Indeed, the morphology of AEB VLPs at the cell membrane visualized by TEM showed that their size and shape was highly variable and generally different to that observed for functional HIV-1 virions, which was consistent with the VLPs having a different sedimentation rate when assessed by rate velocity centrifugation. Given the extensive safety mutations within *gag* and *pol*, it is possible that they facilitated the inappropriate folding of Pr160^Gag-Pol^ polyproteins, such that they were excluded from budding particles, inhibited PR activity, or disordered the Gag lattice within a budded VLP. Ultimately, the presence of the RT domain deletions was found to be the specific cause of incomplete Gag cleavage for the AEB VLPs, and this was consistent with previous observations that RT mutations that destabilise the Pr160^Gag-Pol^ polyprotein multimers can cause premature activation of the PR [72,91]. Therefore, the AEB VLPs were likely assembled from a combination of Pr55^Gag^, p41, and p24 that had undergone partial PR-mediated cleavage within the cell cytoplasm, rather than from uniform Pr55^Gag^ assembly that would normally occur.

The removal of the entire RT domain from the VLP expression cassettes served two purposes. Firstly, it prevented the destabilisation of Gag/Gag-Pol multimers and subsequent aberrant particle assembly. Additionally, the RT functions are not required for viral particle maturation, thus its removal was a safety advantage. The VLP expression vectors that removed the entire RT domain (ΔRT) likely represent the minimal arrangement for VLP vector expressing particles that undergo proteolytic maturation, given that the minimum requirement for HIV-1 particle expression is the Gag polyprotein and the viral PR that is required to cleave Pr55^Gag^ into its functional subunits. This was consistent with particle expression vectors that are employed to produce fourth generation lentiviral vectors, which express Gag polyprotein and a Gag-PR polyprotein from one plasmid, whereas the RT, RNase H, and IN are supplied in *trans* as a Vpr-tagged polyprotein [92]. The particles expressed by these systems need to be able to mediate fusion with the target cell to be effective, which is only possible when proteolytic maturation has occurred [73]. The ΔRT VLPs bearing AD8 Env were able to mediate fusion with CD4+/CCR5+ T cell lines, with only slightly reduced efficiency when compared to wild type 93TH253.3 virions expressing the same Env, demonstrating that they had attained maturity. The level of maturation in ΔRT VLPs would likely allow for VLP-associated Env to laterally translocate on the VLP surface and form Env clusters, as seen on infectious HIV-1 virions [26]. This would need to be confirmed by future experiments to assess the presence of Env foci on the VLP surface by high-resolution imaging techniques, such as stimulated emission depletion microscopy (STED) or stochastic optical reconstruction microscopy (STORM). In the absence of RT, RNase H, and IN, the ΔRT VLPs would be expected to bear an abnormal core structure when compared to that normally seen for infectious HIV-1 virions [93]. This is a possible reason for the reduced fusion efficiency of ΔRT VLPs, since the BlaM enzyme that mediates the reaction was fused to Vpr that is normally found within the core of HIV-1 virions [94]. Nonetheless, the VLPs are intended to be non-infectious particles that present membrane-bound Env for vaccination purposes, so the formation of the electron-dense viral core is not required.

The parental 93TH253.3 strain AE clade virus did not generally demonstrate the complete processing of Gag polyprotein, with variable amounts of the p41 intermediate being detected in addition to p24. The presence of these Gag processing intermediates is undesirable, given that they can reduce the efficiency of viral particle maturation [95]. This could be addressed in future work investigating whether alternative isolates could be utilized, with a particular focus on VLP vectors that express high yields of mature particles, as well as high levels of Env incorporation, given their use as Env immunogens. Such approaches would also need to consider the encapsidation of cellular and viral RNA into the VLPs. This is especially relevant for in vitro-produced VLPs that are injected into vaccine recipients, given that particles with high levels of RNA encapsidation will contain cellular RNA from transformed cell lines that are typically used in recombinant expression systems.

The final mature VLP expression vector (AEB.NCΔRT) bears 70% of the parental 93TH253.3, NL4-3, and AD8 viral sequences. This is slightly above the generally accepted safety standard of retention of no more than two-thirds of the complete virus genome that, when including removal of viral promoters, ensures that there is little possibility of replicative function. The ΔRT mutation within the VLP vector did not remove the mutated RT and RNase H domain nucleic acid sequences that were derived from the original VLP vector, rather a premature stop codon was introduced at the end of the PR domain, such that most of *pol* was untranslated. However, in the unlikely event of the premature stop codon reverting to its wild type sequence, the multiply-mutated non-functional RT and RNase H from the original pVLP vector would be expressed, maintaining safety. Nonetheless, this untranslated region leaves the option open for the further deletion of unnecessary viral sequence from these domains to lower the total viral sequence contained in the VLP vector to be less than the maximum to be considered safe. However, it is critical to consider whether the removal of native HIV-1 mRNA splice acceptor and donor sites within the *pol* sequence or inadvertent introduction of cryptic splice sites would interfere with the virally regulated mRNA expression that is required for these VLP expression vectors. This was clearly demonstrated in the studies here with the introduction of a Tat truncation reducing the amount of Env expression, likely via one or more of the 4a/b/c splice acceptor sites being disrupted [96].

An important aspect of the VLP expression vectors that were developed here was their ability to incorporate membrane-bound Env, given the large number of safety mutations. VLP-associated Env was capable of presenting both gp120- and gp41-specific bNAb epitopes, as demonstrated by VLP ELISA. The VLP-associated Env bound several anti-gp120 bNAbs more efficiently than soluble, monomeric gp120, including b12, PG16, and PGT121, likely due to the presence of trimeric Env on the VLP surface. Enhanced VLP binding as compared to soluble gp120 was also observed for the poorly neutralising antibodies F105 and 17b, indicating the likely presence of improperly folded Env on the VLP membrane, as has been observed on virions [97]. Overall, the mature VLPs displayed a similar antigenic profile to the immature VLPs, indicating that membrane-bound Env was efficiently incorporated into these particles.

In summary, here we describe the design of a single-plasmid expression cassette that is capable of production of mature-form VLPs that are not infectious and safe for use as vaccine immunogens without requiring inactivation. The initial AEB VLP single-plasmid vectors yielded particles, in which Gag was incompletely processed by the viral PR and displayed an aberrant morphology. We found that safety mutations within the RT domain were responsible for the immature phenotype, and that restoring a functional RT domain facilitated the expression of proteolytically mature VLPs. To increase the safety of the VLPs and maintain the mature phenotype, the entire RT domain was deleted and this vector gave rise to VLPs that mimicked the fusibility of infectious HIV-1 virions. The mature VLPs efficiently incorporate membrane-bound Env and display bNAb epitopes within gp120 and gp41. These VLPs, which resemble mature HIV-1 virions, warrant further investigation as potential vaccine immunogens that are able to be delivered either as particles or in a nucleic acid vector to elicit antibody responses against HIV-1 Env.

## Supplementary Materials

The following are available online at https://www.mdpi.com/1999-4915/11/6/507/s1, Figure S1: Analysis of individual and combinations of RT mutations on AEB VLP Gag processing, Table S1: Mutagenic primers used for plasmid construction.
