# Supplementary material for: HIV-1-Based Virus-like Particles that Morphologically Resemble Mature, Infectious HIV-1 Virions
Source: Viruses. 2019 Jun 2;11(6):507. doi: 10.3390/v11060507 (PMC6630479; doi:10.3390/v11060507)
Supplement: Supplementary file 1 [file viruses-11-00507-s001.pdf]

Supplementary data

Table S1. Mutagenic primers used for plasmid construction.

Name	Sequence (5′ to 3′)
Odp.0241	GTCGTAACAACTCCGCCCCATTG
Odp.0479	CATGCTGTCATCATTTCTTCTA
Odp.1067	ATAGAATTCGCTTCTTTCAGTTGTCCTCC
Odp.2856	ATAAAGAATTCATAGTAGCAAAGGAAATAGTAGCC
Odp.2857	ATAAAGAATTCTCATCTAATCCTCATCCTGTCTACC
Odp.3032	ATAAAGCGGCCGCTTTTCGCTTGTACTGGG
Odp.3227	GTACTCTAAATTTCTAAATTAGTCCTATTG
Odp.3228	CAATAGGACTAATTTAGAAATTTAGAGTAC
Odp.3446	TAGGCGGATCCGAACAAGCCCCAGAAGATCAAG
Odp.3447	ATCGACTACTCGAGATCTACCAGCTCCATTCC

Table S2. Ratio of gp120 to p24 concentration of viral particles used in fusion assay (Figure 8B), as determined by gp120 Western blotting and p24 ELISA, respectively. ND, not determined; NA, not applicable (because p24 could not be measured accurately).

Sample	gp120/p24 concentration
AEB.NCΔRT (no BlaM-Vpr)	ND
AEΔB.NCΔRT	0
AEp55B	NA
AEB	18.3
AEB.RT	38.1
AEB.NCΔRT	29.1
AEΔB.NCΔRT + AD8 Env	10.0
93TH293.3 + AD8 Env	5.44

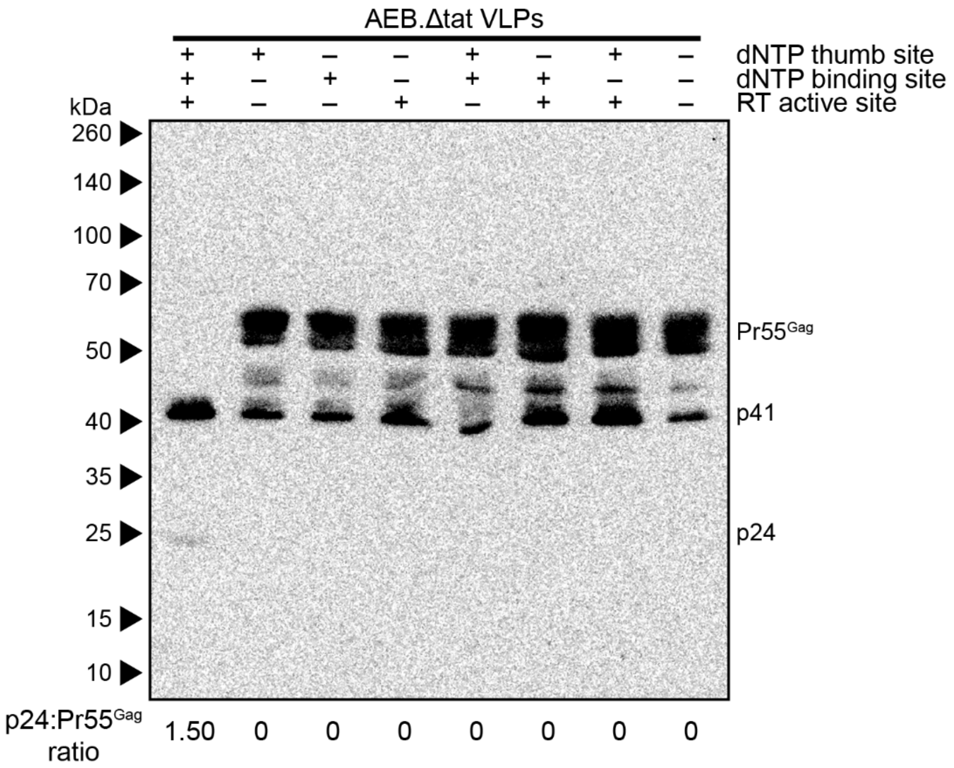

Figure S1. Analysis of individual and combinations of RT mutations on AEB VLP Gag processing. Representative anti-p24 (#24-4) Western blotting on AEB VLPs expressing a non-functional Tat with none, one, or more restorations of the RT domain deletions: dNTP thumb site, dNTP binding site, or RT active site, as indicated. Samples were resolved by 8–16% SDS-PAGE. The positions of Gag polyprotein cleavage products are indicated on the right and protein sizes were indicated on the left by Spectra Multicolour Broad Range Protein Ladder.
